# More than a lack of resources: a mixed-methods assessment of the barriers and facilitators to infection prevention practices in three low-resource maternity units in Malawi

**DOI:** 10.1136/bmjgh-2025-020210

**Published:** 2026-09-15

**Authors:** Jennifer M Riches, Chimwemwe Kachiwaya, Marthe Onrust, Linda Nyondo-Mipando, Catriona Waitt, Abi Merriel, David Lissauer

**Affiliations:** 1 Women's and Children’s Health, University of Liverpool, Liverpool, UK; 2 Maternal and Fetal Health Group, Malawi-Liverpool-Wellcome Trust Clinical Research Programme, Blantyre, Malawi; 3 Kamuzu Central Hospital, Lilongwe, Malawi; 4 Universitair Medisch Centrum Utrecht, Utrecht, Netherlands; 5 Infectious Diseases Institute, Makerere University, Kampala, Uganda; 6 Malawi-Liverpool-Wellcome Trust Clinical Research Programme, Blantyre, Malawi

**Keywords:** Infections, diseases, disorders, injuries, Obstetrics, Global Health, Surgery

## Abstract

**Background:**

Maternal sepsis is a leading cause of maternal mortality and morbidity. Caesarean section is a risk factor for infection, particularly in low-resource settings. This study aimed to identify barriers and facilitators to recommended infection prevention (IP) practices prior to implementation of a complex intervention to improve IP during caesarean section in a low-resource setting.

**Methods:**

This was a mixed methods study conducted in three semi-rural maternity hospitals in Malawi between June and December 2022. Participants were healthcare workers (HCWs) and women undergoing caesarean section. Data collection included case note review, weekly surveys and ethnographic participant observation to assess resource availability for IP and adherence to recommended IP practice. Preliminary data were integrated and used to inform in-depth interviews with HCWs to further explain findings. Inductively coded themes from interviews were mapped to the Communication-Opportunity-Motivation-Behaviour (COM-B)/Theoretical Domains Framework (TDF) model.

**Results:**

We found a lack of resources and suboptimal infrastructure for IP across the facilities, with many basic items available only intermittently. Observation revealed significant gaps in recommended IP practices, including hand hygiene, aseptic technique during vaginal examination, surgical scrubbing and sterile practice in the operating theatre. Scarcity of resources and infrastructure functioned as both direct and indirect barriers to IP due to their impact on staff motivation (reflective and automatic). Having ‘passion’ for patients, regular senior supervision and ‘reminders’ acted as facilitators to recommended IP practice.

**Conclusion:**

Our study demonstrates that adherence to recommended IP practice is determined by more than the availability of resources; the indirect impact of scarcity on staff motivation also played an important role. The study identified further barriers and facilitators to general IP practices, the understanding of which was subsequently used in the selection and development of a complex behaviour change intervention to improve IP practices in the same facilities.

**WHAT IS ALREADY KNOWN ON THIS TOPIC**

- Maternal sepsis is a leading cause of maternal morbidity and mortality, particularly in low-resource settings.
- Caesarean section is the most common surgical procedure worldwide and is a significant risk factor for infection.
- Evidence-based infection prevention strategies can prevent caesarean section-related maternal infection.

**WHAT THIS STUDY ADDS**

- This study identified barriers to recommended infection prevention practice by healthcare workers in low-resource maternity settings.
- It provides information about reasons for the ‘know-do’ gap in infection prevention practice.

**HOW THIS STUDY MIGHT AFFECT RESEARCH, PRACTICE OR POLICY**

- This study underlines the importance of consistency in supply of basic resources for infection prevention, of close mentorship and supervision of healthcare workers, and highlights the importance of understanding the context for intervention selection and implementation.

## Background

 Global maternal mortality ratios remain unacceptably high.^1^ With increased uptake of facility-based births,^2^ the need for improved quality of care across facilities in low-resource settings has been brought into focus.^3^ This is particularly poignant in relation to infection-related maternal deaths as adherence to recommended infection prevention (IP) practice by healthcare workers (HCW) is key to preventing healthcare-associated infections such as those occurring following caesarean section.^4^

Caesarean section rates are increasing across all global regions.^5^ Although many low-resource settings remain underserved by this potentially life-saving surgery,^5 6^ it is in such contexts that caesarean section is more frequently associated with maternal morbidity and mortality.^7^ A global meta-analysis estimated that 22% of maternal deaths following caesarean section in low- and middle-income countries are caused by sepsis.^7^ Infection following caesarean section may cause severe maternal infection and/or maternal sepsis, leading to the need for prolonged hospitalisation, repeat surgical procedures, emergency hysterectomy (with permanent infertility), and death.^8–10^

Malawi is a low-income country in Southeastern Africa. Maternal mortality is estimated at 381 per 100 000 livebirths^1^ with infection the leading cause of maternal death.^11^ Recent analysis revealed that over 50% of maternal deaths occurred after caesarean section, and women undergoing caesarean section were more than twice as likely to die of pregnancy-related infection than those delivering vaginally.^12^

There is global precedent for reduction in maternal infection, achieved primarily through improved IP. Evidence-based strategies for prevention of caesarean section-related infections exist; general IP measures such as surgical sterility, hand hygiene and surgical scrubbing,^4 13^ as well as caesarean-specific strategies including antibiotic prophylaxis, abdominal skin preparation and vaginal preparation with antiseptic^14^ are recommended by the WHO. Single dose antibiotic prophylaxis using a first-generation cephalosporin or penicillin is recommended for all women undergoing emergency or planned caesarean section.^14 15^ Vaginal preparation with antiseptic is recommended immediately prior to skin incision as it has been shown to reduce the incidence of post-caesarean endometritis from 7.1% to 3.1% (RR [Relative risk] 0.41, 95% CI 0.29 to 0.58)^14 16^ likely through reduction in pathogens causing ascending infection. Finally, application of antiseptic to the abdominal skin reduces the number of microbes and the incidence of postoperative infection.^14 17^

Despite the recommended use of evidence-based interventions to reduce infection related to caesarean section, knowledge gaps remain regarding the extent to which they are implemented, feasible and sustainable in low-resource settings. This study took place prior to the implementation of a complex intervention to improve IP at the time of caesarean section. Our aim in this baseline study was to better understand the context for implementation and inform intervention selection and development by identifying the barriers and facilitators to IP practices in low-resource maternity settings.

As our intervention required a change in HCW IP practices, we selected the Capability-Opportunity-Motivation-Behaviour (COM-B) model of behaviour change to inform our study, including the baseline component described in this paper. COM-B conceptualises behaviour as a ‘system’ in which capability, opportunity and motivation interact to generate behaviour.^15^ Capability is the individual’s psychological and physical ability to engage in the behaviour/practice in question.^15^ Motivation encompasses all cognitive processes that inspire, drive and direct behaviour and includes both automatic (eg, unconscious habit) and reflective (eg, analytical decision-making).^15^ Opportunity describes all the factors that lie outside the individual that facilitate the behaviour and includes both social and environmental factors.^15^ Each of the components of behaviour in the COM-B model is further broken down and described by the ‘Theoretical Domains Framework’ (TDF), derived from theories pertaining to implementation of interventions. See online supplemental file S1 for further information.^16–18^

## Methods

### Study design

This was a mixed methods observational study conducted during the baseline phase of a complex intervention study from June 2022 to December 2022.

### Study setting

The study was conducted at three semi-rural government healthcare facilities in the Central Region of Malawi. One was a district (second level) hospital (A), the others were community (first level) hospitals (B and C). All three provided caesarean section and met the definition of low-resource settings (including financial shortages, underdeveloped infrastructure, human resource limitations and suboptimal healthcare service delivery),^19^ as assessed during previous projects carried out at these sites.^20–22^

### Participants

Eligible participants included all maternity HCWs at the study sites and women undergoing caesarean section, including both planned and emergency procedures. Women who had undergone caesarean section were identified by staff working in the maternity department who alerted data collection officers (DOs) who attended as soon as the woman was deemed clinically stable. Patients were provided with an information leaflet (read aloud if necessary) in Chichewa and formal written consent was obtained (online supplemental file S2). If they wished to proceed, they signed (or thumb-printed) a consent form.

### Data collection and analysis

**Figure 1:**
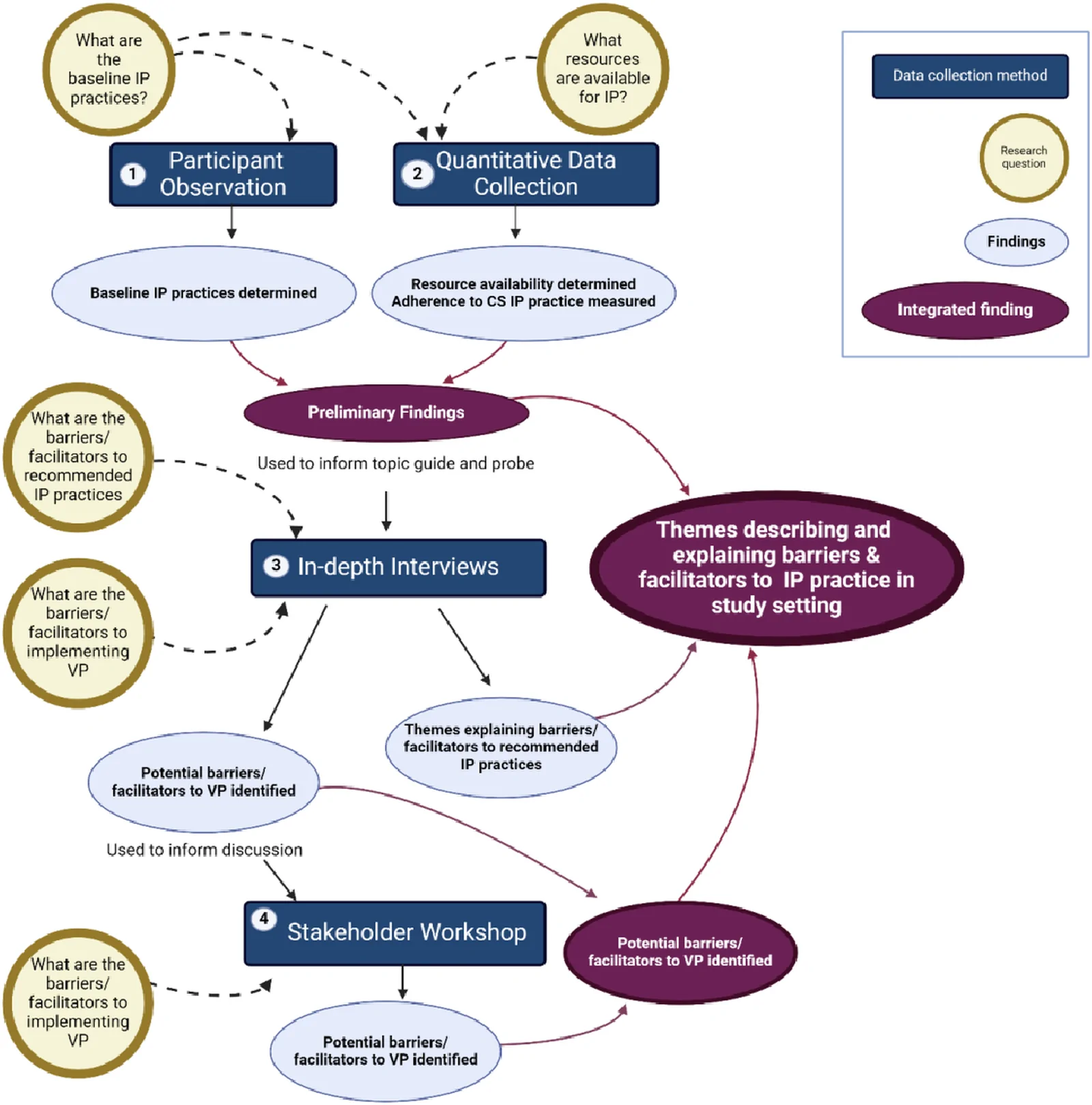
Data collection methods and their integration to describe barriers and facilitators to infection prevention practice in three low-resource maternity settings in Malawi. CS, caesarean section; IP, infection prevention; VP, vaginal preparation.

Key research questions were developed to address the overall aim of the study (figure 1). Exploratory methods of data collection included quantitative methods (survey and case note review) and ethnographic participant observation. These were integrated at the analysis stage, before informing in-depth interviews with HCWs (figure 1). A stakeholder workshop was later held to explore barriers and facilitators to a potential intervention to improve IP at the time of caesarean section, though reporting of its findings is beyond the scope of this paper.

#### Quantitative data collection and analysis

Quantitative data was collected by DOs; HCWs trained in the study protocol who joined the study team. They completed case report forms (online supplemental file S4) using a tablet-based application (OpenDataKit V.1.21.0). Study team members visited the facility regularly to verify data entered and to troubleshoot. DOs were able to contact the study team with queries.

The objective of quantitative data collection was to assess resource availability for IP practices and baseline adherence to recommended evidence-based practices for the prevention of infection at caesarean section. This was achieved through weekly facility-reported surveys of availability of basic resources for IP including soap, alcohol gel, antibiotics, gloves, gauze, antiseptic, sterile delivery packs and caesarean section packs as well as running water and electricity (online supplemental file S5). DOs completed a weekly survey regarding resource availability relevant to IP by visiting relevant areas of the maternity department (labour ward, theatre) to determine what the availability of resources had been over the preceding week. DOs rated resources as available ‘every day’, ‘most days’, ‘some days’ or ‘never’.

DOs retrospectively reviewed case notes to assess adherence to recommended IP practices at the time of caesarean section. The standard for IP practices was taken from WHO recommendations^4 14^ and the Malawi Standard Treatment Guidelines^23^ and included assessing whether the woman received antibiotic prophylaxis, and whether skin preparation and vaginal preparation with antiseptic were conducted prior to skin incision.

Quantitative data analysis was carried out using descriptive statistics in R (V.4.2.3) with calculation of 95% CIs for proportions and IQRs for medians. Adherence to WHO guidelines for IP at caesarean section was calculated using proportions and CIs. Heat maps were created showing week-by-week resource availability.

#### Participant observation data collection and analysis

Participant observation is a form of ethnographic research.^24^ It has been valuable in understanding the delivery of healthcare and how organisations and individuals function within their working environment.^25^ We used participant observation to gain understanding of the context for IP in the study setting. It was important that the daily lives of HCWs, their tasks, how they used resources and the challenges they faced be appreciated prior to implementation of an intervention. We observed baseline adherence to recommended IP practices, availability and use of resources for IP.

Data was collected by JMR (British female researcher and obstetric resident doctor) and CK (Malawian nurse-midwife and study coordinator) according to a framework developed using national and international guidance on IP in maternity and surgical settings.^4 13 14 23 26–28^ Elements from this guidance pertinent to preventing infection for women undergoing caesarean section were selected as domains constructing the framework. These included hand hygiene (on wards and in theatre), vaginal examination (most women undergoing caesarean section in this setting do so after undergoing vaginal examination in labour), antibiotic prophylaxis, surgical hand preparation (scrubbing) prior to caesarean section, safe surgical practice and surgical technique and postoperative care of caesarean section patients. Patient hygiene was added as an additional domain in the early stages of observation as it emerged as a possible risk factor for infection, and national guidance regarding this was available. Each domain became an area of focus for observation in the facility.

Observation commenced before quantitative data collection to avoid staff having detailed awareness of what the study team were observing and thus modifying their behaviour.^29^ However, staff were aware that we were looking to determine strategies to improve infection prevention and to assess resources available for infection prevention. However, we did not advise staff of which particular infection prevention behaviours we were interested in. Observation was initially conducted during daytime hours from Monday to Friday at each facility, then followed up with visits at least once per week and observations made while on site to conduct other activities. Observation took place primarily on the labour ward and in the operating theatre and included unstructured discussions and participant-led conversations. The study team made notes during and following visits to facilities.

A framework analysis of participant observation data was undertaken, using the domains outlined. Field notes and transcriptions of verbal debrief conversations between CK and JMR were transcribed and imported into QSR NVivo V.12 Plus software. Data were then ‘coded’ according to the deductively generated categories outlined above.

Following analysis, preliminary findings from participant observation were integrated with quantitative baseline findings to describe resource availability and adherence to baseline IP practices and to inform the development of a topic guide for in-depth interviews (see below).

#### In-depth interview data collection and analysis

Baseline interviews were used to explain preliminary findings and explore barriers and facilitators to recommended IP practice. An interview guide was developed using a ‘vignette’ (clinical story) based on participant observations and used to allow interviewees to comment on hypothetical scenarios, explaining what a third person might say, do or think in a particular situation (online supplemental files S6 and S7). Vignettes are particularly useful to gather interviewees’ beliefs or perceptions about sensitive topics where they might not feel comfortable to talk about their own personal thoughts, beliefs or actions.^30 31^ The vignettes used told the story of a woman attending a healthcare facility in labour, undergoing caesarean section and developing an infection. Participants were asked to comment on the IP practices of HCWs during the woman’s journey through the facility. Two different versions of the vignette were used: one for staff working in theatre and one for staff working on labour ward.

In-depth interviews were conducted at all study sites using purposive sampling to capture different cadres of maternity staff and continuing until data saturation. At Hospital A, recruitment was opportunistic, with the researcher based in a clinical area and inviting participants to interview whenever they were available. At Hospitals B and C, DOs invited staff to participate in advance, based on their availability and cadre. All interviews were conducted by JMR, in English (the predominant language used in healthcare), although if it was felt that the participant might struggle to express themselves, a Chichewa-speaker would be present to translate where necessary. All participating staff were given a patient information leaflet and provided written consent (online supplemental file S3). Their anonymity was guaranteed.

Interviews were audio-recorded and transcribed prior to being imported into QSR NVivo V.12 Plus software prior to reflexive thematic analysis. Codes for in-depth interview data were inductively generated from the data itself (JMR). Codes were linked, grouped and summarised as themes (JMR), which were then reviewed and agreed by CK, AM and LN-M. Themes were then mapped to The COM-B model and TDF (online supplemental files S1 and S2) to gain a deeper understanding of the barriers and facilitators to IP practices based on established and evidence-based behaviour change theory.

## Results

### Quantitative findings

Quantitative data were collected over a 14-week period from September to December 2022. Of the 589 women who underwent caesarean sections across all facilities during the observation period, 577 (98.0%) were enrolled in the study. Over 90% of women undergoing caesarean section received antibiotic prophylaxis and over 95% received skin preparation with antiseptic (table 1). Vaginal preparation with antiseptic was carried out in less than 2% of cases, all of which occurred following a training event for the implementation of the complex intervention study which followed.

**Table 1:** Adherence to WHO-recommended caesarean section-specific infection prevention practices

Recommended practice	Adherence n=577 (% of cases)	95% CI
Received antibiotic prophylaxis	520 (90.1%)	87% to 92%
Skin preparation with antiseptic	575 (99.6%)	98.6% to 99.9%
Vaginal preparation with antiseptic	8 (1.4%)	0.6% to 2.8%

Data regarding resource availability was collected for 11 of 14 weeks at Hospital A and for all 14 weeks at Hospitals B and C. Heatmaps showing resource availability for IP available at each facility in both labour ward and theatre areas are found in online supplemental file S8. Supply of resources for IP was intermittent across all facilities. For example, Hospital B had limited availability of both first-line antibiotics for caesarean section prophylaxis throughout the observed period. Both hospitals B and C had limited availability of soap and alcohol gel in labour ward. Hospital C lacked running water in labour ward and theatre on a regular basis.

### Participant observation findings

Observation commenced in June 2022 and was initially conducted for intensive week-long periods at each facility, then followed up with visits at least once per week, and observations made while on site to conduct other study activities. Observation took place on labour ward and in the operating theatre; there was less activity for observation on postnatal wards. All areas of the maternity wards featuring in the journey of a woman undergoing caesarean section and all HCWs she might interact with (eg, nursing/midwifery staff, clinicians, theatre staff, nursing attendants) were covered. Table 2 provides the findings of participant observation and integrates them with quantitative results under the domains of the framework. The findings of participant observation are summarised according to the domains of the adopted framework in online supplemental file S9.

**Table 2:** Integration of quantitative and participant observation data under domains of framework

Domain	Quantitative findings	Observation findings
Hand hygiene (HH**)**	Intermittent availability of running water. Intermittent supply of soap. Intermittent supply of alcohol gel. Consistent supply of gloves.	Improvised buckets to provide running water were available, but sometimes inconveniently located. Staff were not observed to perform HH before or after patient interaction. Alcohol gel was shared between staff and clinical areas, and frequently moved between locations. HH not performed even if resources were available and correctly located.
Vaginal examination (VE)	Intermittent availability of antiseptic. Intermittent availability of gauze. Consistent supply of gloves. Intermittent supply of alcohol gel/soap for hand hygiene.	HH never performed prior to preparing equipment or conducting VE. Gloves always used to conduct examination. Antiseptic rarely used prior to VE, even when available. Aseptic non-touch technique poorly observed with handling of sterile gauze and contamination of equipment by placing on bed/patient clothing prior to use. VEs carried out more frequently than recommended.
Antibiotic prophylaxis	Over 90% of women received prophylactic antibiotics. Most received prophylaxis preprocedure. 73% received prophylaxis 15–60 mins before incision. Benzylpenicillin most often used. Around half of women received one dose only and half received extended courses.	Standard practice for LW midwives to give prophylaxis as soon as the decision for CS made, often hours ahead of procedure. Most women received extended courses of intravenous antibiotics following caesarean section. Women often missed receiving antibiotics due to lack of equipment such as syringes. Despite low resource availability demonstrated by surveys, adherence to prophylaxis practice was high because women were asked to buy their own.
Surgical hand preparation	Running water was intermittently available for scrubbing. Soap was consistently available in theatre at two sites, and almost always available at the third site. Antiseptic scrub was never available. Sterile gloves were not available every day at any of the sites.	Buckets were used to provide running water for scrubbing. Buckets and sinks did not provide adequate space to rinse elbows vertically under taps. Non-touch taps were not available at any site. Duration of scrubbing was less than that recommended. Staff did not make use of colleagues to turn off taps, and often used their scrubbed hands instead.
Skin preparation with antiseptic	Carried out in >99% of cases. Chlorhexidine and povidone-iodine most often used.	This practice was ingrained; it would be unthinkable for staff to proceed to CS without performing. Two antiseptic agents were often used sequentially. Application was often haphazard and involved risk of desterilisation of the operator/wound site.
Vaginal preparation	Good availability of antiseptic and gauze on a weekly basis.	Not part of routine practice at any of the facilities. Resources to implement VP were routinely available.
Safe surgical practice and surgical technique	Two sites had availability of sterile instrument packs for CS ‘every day’ during the baseline period. One site did not have availability ‘every day’ for four of the weeks. Sterile drapes and gowns were intermittently available across all sites. Most CS were carried out as emergencies. More than half of these at ≥4 cm dilated. 25% of CS were carried out when the woman was fully dilated. 96% were carried out by clinical officers	Shortage of instruments meant that there was frequently a shortage of packs at Hospital A, and a shortage of certain instruments within packs at B and C. CS patients at Hospital A had to be transferred to the central hospital, more than 1 hour away when packs were not available. Gowns and drapes were often in short supply, and those that were available were often threadbare with obvious defects. Patient gowns not available, *chitenje* used to cover parts of patient instead. Sterility was often not maintained in the operating theatre; scrubbed staff were frequently observed being contaminated, but proceeding with surgery and in turn, staff who were not scrubbed often contaminated sterile fields and equipment. Resources necessary for theatre cases were conserved by staff and prioritised to the theatre environment. CS carried out by junior clinicians without easy access to senior support. Many difficult fetal extractions, potentially leading to trauma of maternal tissues and increased risk of infection. Preference among surgeons for use of chromic catgut sutures for closure, and habitual conserving of suture material. Lack of attention to haemostasis. Surgical checklist not used, and no routine practice of counting swabs and instruments.

CS, caesarean section; HH, Hand hygiene; LW, labour ward; VE, Vaginal examination; VP, vaginal preparation.

### In-depth interview findings

In-depth interviews were conducted with HCWs working in labour ward and theatre. 17 staff from across facilities participated in baseline interviews (online supplemental file S10).

#### Theme 1: healthcare workers have knowledge about recommended infection prevention practices, but it is normal not to perform them

**Figure 2:**
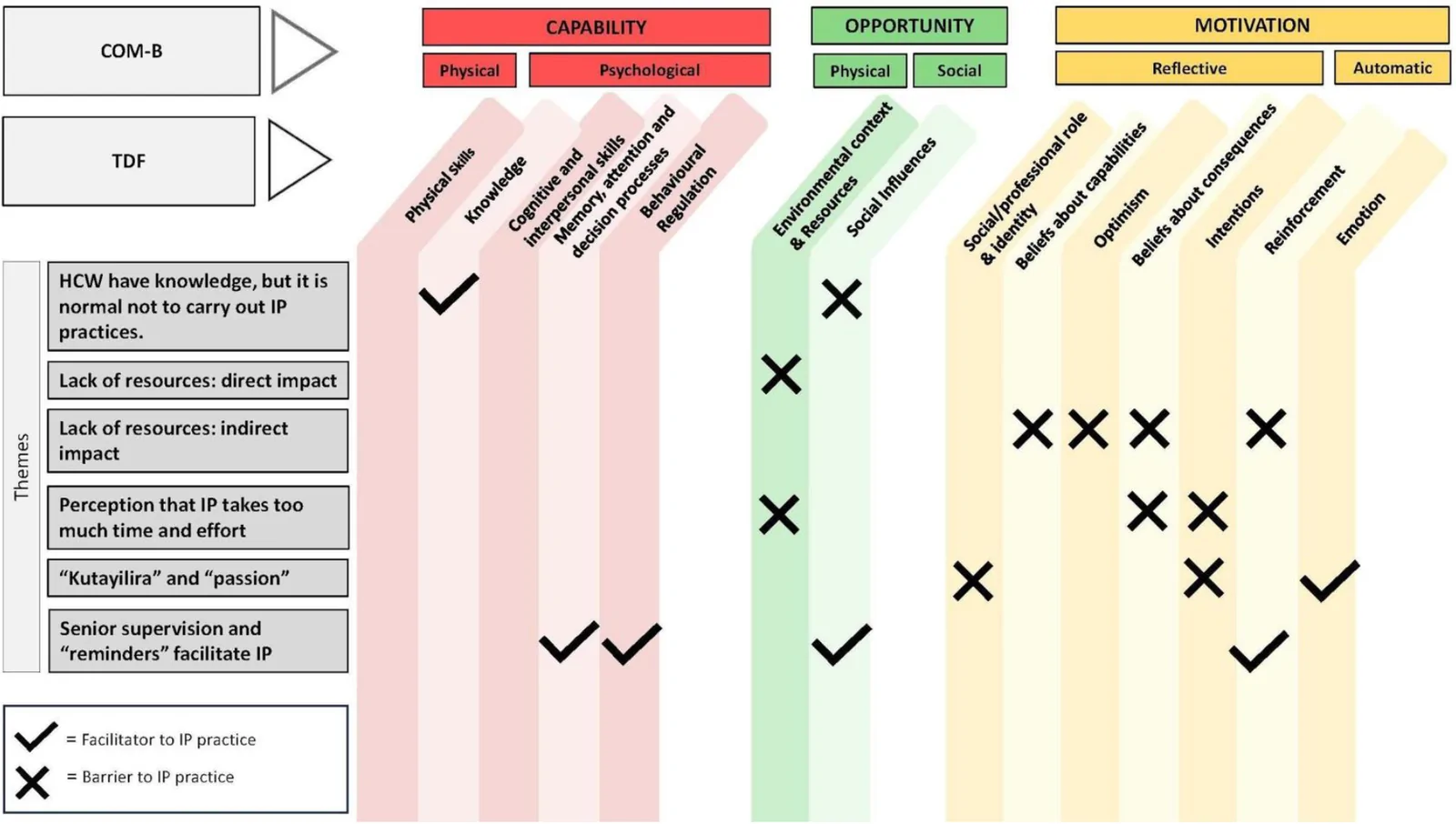
Barriers and facilitators to infection prevention in three low-resource maternity settings in Malawi. COM-B, Capability-Opportunity-Motivation-Behaviour model; HCW, healthcare worker; IP, infection prevention; TDF, Theoretical Domains Framework.

With the exception of VP, HCWs could explain in detail IP practices that should be undertaken to avoid maternal infection. When challenged with data from participant observation, participants acknowledged that it was normal for these practices NOT to be performed, even when resources are available. They explained that it is normal for HCWs to take ‘shortcuts’ in IP practice (see table 3, Q1/2). This theme mapped to psychological capability and social opportunity in the COM-B/TDF framework (figure 2).

**Table 3:** Quotes illustrating key themes emerging from qualitative interviews with healthcare workers regarding barriers and facilitators to infection prevention practices

Theme	Quote	Participant quotes
Healthcare workers have knowledge about recommended infection prevention practices, but it is normal not to perform them	Q1	“Usually, some people they just miss some steps for doing that examination but usually it is supposed to be like that. You [should] do six swabs technique, then you do your examination… It’s just a matter of maybe they’re used to that, but it supposed not to be like that. Some they will just think that this will be nice for me to just do like this, but in real sense it doesn’t mean to be done like that.”
	Q2	Interviewer: “So, if you have the resources that you need, if-if Ernest has resources that he needs, then every time will he do it correctly?” Respondent: “Yes”. Interviewer: “Every time? 100 percent?… Because that’s not what I have observed”. Respondent: “(Laughs) Mm-hmm… that’s … in ideal settings, we have to do that… but because of some negligence you know… we don’t.” (P9)
Lack of resources acts as a direct barrier to infection prevention practices	Q3	“One thing is the scrubbing area… you cannot do the real scrubbing itself. The other thing, is the water, you usually use the bucket most of the times ‘cause we don’t have running water throughout… It’s more like you are bathing, not the real scrubbing itself-cause the tap is down, it’s very low that you cannot even try to bend into, to get under.” (P2)
	Q4	“So, the resources we have, we use them only for important things… chlorhexidine we don’t have so we don’t bother, most of the times we use [tap] water to do 6 swabs… then after delivery we use that cotton to expel clots, we don’t use it for 6 swabs [vaginal examination], we use it after delivery… sometimes we even use cloth of a woman to expel the clots. So, we reserve it [the resources] only for important things.” (P14)
	Q5	“Sometimes it [not following recommended practice] can be due to workload, because we reach a point of working 2 qualified staff at a shift so you are covering labour ward, postnatal and kangaroo mother care. So, sometimes you can have almost 18–20 deliveries that day… you are 2, you need to discharge from postnatal, so I think workload can contribute to that…” (P11)
Lack of resources acts as an indirect barrier to infection prevention practices	Q6	Participant: “Maybe he has forgot” Interviewer: “He has forgotten to wash the hands?” Participant: “Yes, because water is there [today], but there are sometimes water is not there, we don’t bother to wash hands.” (P14)
	Q7	“I haven’t seen one performing a proper scrubbing just because of the setup. We can’t expect someone to scrub using that bucket for [the required time]… no… We don't have a proper scrubbing sink, so the clinicians here are just used to short cuts.” (Participant was then asked if someone would still do it this way if all the necessary resources and equipment were available, she said:) “Yes, exactly they will still do it this way… because they will get used to it, because it will be normal for them.” (P3)
	Q8	“Sometimes because we may have erratic supply of resources, so you can have maybe sometimes the time when you don’t have the face mask or you don’t have the cap. So, people feel that if I did the procedure last time without a cap because the things were not there, this time they are there but they just think that aah it’s okay because we did that last time without the resources” (P15)
	Q9	“…So you see that whatever I am trying to do, it is almost nothing if I don’t have that, that, or that. So without resources, working in maternity, in theatre it’s a very, very big problem, which people get discouraged… because okay, even if I do A, B, C, D but if I don’t have A, B, C, D its equates to nothing. So, you plan to go to theatre when you don’t have antibiotics, how do you think this woman will heal the wound? It will be a problem. They end up coming back a week later that’s for sure because you did not give them enough or necessary resources, so they come back, they end up having TAH [hysterectomy] so without resources it’s very, very challenging and demoralizing” (P15)
A perception that infection prevention takes time and effort acts as a barrier to recommended infection prevention practices	Q10	“As I said, sometimes we do things in hurry…pressure of work, there is much workload, so we remember to wash hands after everything is okay. But if they can call me right now, I will just wear gloves without washing hands and do the required thing.” (P14)
	Q11	“…sometimes you might find it very difficult … you say ‘Aah…, this one, I can just do it without [infection prevention]’, but the resources are there. Or sometimes, maybe you are only one working in the labour ward and the ward is very busy… so you might find it very hard to do all the standards of IP [infection prevention]” (P11)
‘Kutayilira’ and ‘passion’ for patients affects IP practice	Q12	“I can say that maybe we are not used to it [performing standard infection prevention practices performing standard infection prevention practices]; you know in Malawi, there is a tendency of… we tend to neglect common, normal things which are done, which are supposed to be done normally… but we tend to sideline them, and we are used to short cuts and improvising.” (P3)
	Q13	“Negligence is seen… the resources are there but they don’t want to do the thing… Because, maybe they might not see it’s necessary for them to do that… or sometimes they might say ‘Aah, this one is not my relative, so no need to follow all the procedures… She is not related to me, so why should be, doing all the procedures, I just need to do shortcuts…’” (P11)
	Q14	“Yeah, so that goes with attitude. Aah, you know… (laughs)… the first thing that you have to do is… like, like you need to have passion for the patients because if you have passion for the patients, you don’t have problem with infection prevention… you just have to treat each and every patient like you are treating your wife or you treating your sister.” (P13)
	Q15	“Is it possible? It’s very possible, it just have to come from your heart… you have to feel that you can do it. Because you may have resources, but if you don’t have that heart, you can't do it… you have to have that heart that you can do the things… the attitude itself.” (P15)
Senior supervision and ‘reminders’ facilitate good infection prevention practice	Q16	“…if there is no one to follow us up on what we are doing we end up neglecting that [the correct procedures], because if there is no one supervising us, it will be hard for us to follow the standard procedure. But I think if the top people would go to check what is happening there, I think these are the things that can be corrected and they are simple things, but we neglect them.” (P3)
	Q17	“That’s their attitude, so they have to be reminded, we have to have somebody in theatre who is in charge who can reprimand these people, who can now and again tell them that once we are in theatre, ‘please let’s follow this thing.’” (P15)
	Q18	“So, if the theatre nurse is [younger and] less experienced or maybe she is afraid to caution the one doing the procedure they shrink; meaning to say: if this man has said this then I just need to bind with it, which should not be the case” (P4)

IP, infection prevention; P, Participant; Q, Quote; TAH, total abdominal hysterectomy.

#### Theme 2: lack of resources acts as a direct barrier to infection prevention practices

Participants explained that not having the correct resources (antibiotics, cotton, sterile gauze, syringes, antiseptic solution) was a barrier to performing recommended practice (table 3, Q3/4/5). Poor facility infrastructure (lack of water/electricity, sinks for scrubbing prior to theatre, sterilisation equipment) was also cited as a barrier. Participants described conserving resources due to the threat of unpredictable stock-outs; explaining why some recommendations were not carried out even when resources were available. HCWs mentioned lack of human resources as a barrier, with high workload given as a reason for not following IP protocols. This theme mapped to physical opportunity in the COM-B/TDF framework (figure 2).

#### Theme 3: lack of resources acts as an indirect barrier to infection prevention practices

Erratic availability of resources was described to negatively affect reinforcement, detracting from subconscious habit formation. Habits for good IP practice are not formed due to inconsistencies in the availability of resources, such that when resources *are* available, the practice is still not performed due to absence of automatic motivation (table 3, Q6/7 and figure 2). Staff described consciously deciding *not* to follow recommended practice based on past experiences of being unable to perform them due to lack of resources, mapping to reflective motivation in the COM-B/TDF (figure 2). This worked through two different mechanisms—optimism (‘nothing bad will happen because it didn’t last time’, see table 3, Q8) and fatalism (‘nothing I do personally can prevent infection’, see table 3, Q9).

#### Theme 4: a perception that infection prevention takes time and effort acts as a barrier to recommended infection prevention practices

A perception existed among HCWs that IP practices take too long, and that in an emergency, they should not be prioritised (table 3, Q10). Other staff commented that they might not perform IP practices to the recommended standard if their workload was heavy, or during a night shift, due to both lack of time and desire to conserve energy/effort (by not performing what were seen as optional practices) until it was most needed (eg, during an emergency) (table 3, Q11). This theme mapped to physical opportunity and reflective motivation in the COM-B/TDF (figure 2).

#### Theme 5: ‘Kutayilira’ and ‘passion’ for patients affects IP practice

Participants described that even with knowledge of how and why to perform infection prevention practices, with ideal resources available and with adequate staffing for workload, many HCWs still did not carry out practices as indicated. Most participants stated that this was ‘just an attitude’, blaming what they referred to as ‘negligence’ in English, or ‘kutayilira’ in Chichewa, meaning to do things without care and attention or ‘without passion’ (table 3, Q12/13). Conversely, some participants also spoke of having ‘passion’ or ‘a heart’ as facilitating good IP practice (table 3, Q14/15). This theme mapped to reflective motivation in the COM-B/TDF framework (figure 2).

#### Theme 6: senior supervision and ‘reminders’ facilitate good infection prevention practice

Participants mentioned the need for improved senior supervision, mentoring and regular ‘reminders’ to perform recommended practices (table 3, Q16). Providing ‘reminders’ was specifically mentioned as a means of overcoming attitudes of ‘kutayilira’ (table 3, Q17). However, ‘reminders’ needed to come from those higher up the hierarchy (table 3, Q18). Age, cadre, working experience and gender factored in where someone appeared in the hierarchy of the team. Some (more senior) participants advocated giving reminders as a means of reinforcing the desired practices (‘automatic motivation’) to support cognitive memory. At other times, ‘reminders’ were cited as necessary to overcome an attitudinal barrier. Staff were asking for ‘reminders’ to support behaviour regulation, expressing a need to be held accountable by those higher in authority. This theme mapped to psychological capability, social opportunity, and automatic motivation (figure 2).

Findings from all baseline data collection methods were integrated and mapped to the COM-B/TDF model to develop an understanding of adherence to recommended IP practices and barriers and facilitators to this in the study setting (figure 2).

## Discussion

In this study we used mixed methods of data collection integrated to form a comprehensive picture of adherence to recommended IP practice, exploring barriers and facilitators to recommended practice in three low-resource maternity facilities in Malawi. Significant gaps were identified, including poor hand hygiene, suboptimal aseptic technique and inadequate surgical scrubbing and sterility. Whilst antibiotic prophylaxis and skin preparation were routinely implemented, vaginal cleansing with antiseptic had not been adopted at any of the facilities, though resources were available for its implementation.

We found that erratic supply of resources and poor infrastructure were both directly and indirectly linked to IP practice. HCWs demonstrated knowledge of recommended IP practice (a facilitator), but non-adherence was normalised, undermining social opportunity for best practice. Lack of resources hindered automatic and reflective motivation; habit formation and HCW beliefs about consequences, optimism and their personal and collective capability to prevent infection were negatively impacted. Further barriers to recommended IP practices included a mindset of ‘kutayilira’, described as an attitude of doing things without care and attention (‘like riding a bike without holding the handlebars’). The reasons for this attitude of ‘kutayilira’ among HCWs were difficult to pin down, and it is possible that reasons for this attitude overlap with other themes identified from in-depth interviews. For example, does ‘kutayilira’ develop because staff are burnt out, demoralised and disempowered due to the context in which they work? Conversely, we found that ‘passion’ for patients acts as a facilitator to good IP practice, mediated through automatic motivation (emotion). Senior supervision and ‘reminders’ also facilitated good IP practice through improving psychological capability (memory, attention and decision processes, and behavioural regulation), promoting social opportunity and providing automatic motivation (reinforcement).

Two domains of the TDF (*Cognitive and Interpersonal Skills* and *Physical Skills*) were not represented in the mapped barriers and facilitators. In relation to *physical skills*, this likely reflects challenges observing physical proficiency in an environment where recommended practices were inconsistently performed, rather than a true lack of capability. Given the content of standard clinical training and because the required procedures were relatively simple, it is reasonable to assume that HCWs possessed the necessary physical skills to complete relevant IP practices. *Cognitive and Interpersonal Skills* may also have been under-represented because infection prevention practices were largely procedural rather than reliant on complex reasoning or interpersonal coordination. Their absence may partly reflect methodological limitations; unless cognitive or interpersonal processes were verbalised during interviews or visibly enacted during observation, they were unlikely to be captured. Interview participants may also have felt more able to discuss external constraints (resources, norms, workload) than their own internal reasoning or interactional dynamics. Further studies might better assess this by using simulation or asking participants to narrate their activities and thought processes. Overall, our findings suggest that the barriers to good IP practice in this setting were related more closely to opportunity and motivation of HCWs than capability.

Strengths of this study include the participation of three maternity departments in data collection, lending validity to findings observed across facilities. Furthermore, the study was set in a district (second level) hospital and two community (first level) hospitals, rather than central teaching hospitals. Our findings are therefore relevant to the experience of ‘typical’ facilities in Malawi and other low-resource settings, where most healthcare is delivered outside of urban centres. The use of mixed methods of data collection enabled triangulation and cross-validation of data collected using different methods. A recognised, evidence-based model to explain and understand HCW practices was used (COM-B/TDF), strengthening and increasing the potential for generalisability of findings between this and similar studies, and directly informing the selection of intervention strategies used in the implementation study which followed.

Limitations include a geographical focus around Lilongwe, and exclusion of private facilities (which provide 13% of maternity care).^32^ Another possible limitation of the study was the employment of HCWs from the facilities as data collection officers, introducing the possibility of bias. To mitigate, study team members regularly visited to cross-check data entry with patient notes and verify resource availability. Data collection regarding resource availability at Hospital A was incomplete, limiting findings from this site. An additional limitation was that observations were made only during daytime working hours between Monday and Friday. It is known that adherence to IP practice is often worse during evenings, night-times and weekend shifts. However, we felt that observations made during daytime practice gave an adequate starting point in appreciation of the context for IP in this setting. Out of hours practice was explored in qualitative interviews with staff to inform our findings. Furthermore, we accept that data collection using participant observation is vulnerable to ‘The Hawthorne effect’, whereby being observed affects the behaviour of participants.^29^ Given the poor adherence to IP practices we observed, we do not believe that this phenomenon had a significant impact on the data collection. Methodological limitations in assessing two of the domains of the TDF have been discussed above.

The lack of resources for basic IP practice found in our study was not surprising; this is a recognised problem in many settings worldwide. In a global report, the WHO noted gaps in the resources and infrastructure for IPC across all regions.^33^ Previous work in Malawi has determined water, sanitation and hygiene (WASH) facilities in hospitals to be substandard,^34^ and the most recent assessment confirmed that inadequate and erratic availability of beds, electricity and WASH is a challenge nationally.^32^ However, this assessment found that 93% of facilities had soap available in maternity areas,^32^ contrasting with our finding that soap was only intermittently available, particularly at Hospitals B and C.

The published literature on IP to prevent healthcare associated infection in low-resource settings primarily focuses on implementation programmes rather than quantifying resource fluctuations. Our findings are novel in describing how erratic availability influences daily practice, challenging the assumption that when supplies exist in a general context of scarcity, they are automatically used. Furthermore, the causes of stock-outs often remained unclear, even after extended observation.

The existence of a ‘know-do’ gap—HCWs knowing but not performing IP practices—is well-recognised.^35^ Even when evidence of benefit is strong, HCWs in all settings may struggle to implement IP practice. There is a paucity of literature (particularly of a qualitative nature) from low-resource settings examining the implementation of IP recommendations. However, similar barriers to IP practice have been described in Uganda^36^; shortage of HCWs and finances, intermittent supply of water, IP materials and equipment, lack of support and supervision, negative attitudes and lack of training and orientation.^36^ Facilitators included management support, the presence of an infection prevention and control committee, visual reminders, supportive supervision, continuing medical education sessions, an organised environment, teamwork and cross-organisational collaboration.^36^ However, extensive exploration of these barriers and facilitators for closer comparison with our study was not available in the published manuscript. Previous studies from the Malawian context have noted similar findings to our study in terms of the ‘know-do’ gap and some of its influencing factors. Merriel *et al*^20^ similarly observed that HCWs knew correct procedures but often omitted them, partly due to poor supervision and frequent senior staff absences.

The literature on the implementation of hand hygiene recommendations and barriers and facilitators to this practice is more extensive than that exploring other areas of IP. The practice of hand hygiene was noted to be a particular challenge during our study, with no member of staff observed to perform hand hygiene prior to patient contact, including when performing intimate examinations. The finding that HCWs struggle to implement hand hygiene is not novel and has long been a recognised challenge in IP across all global settings; a systematic review of studies from high-income countries found hand hygiene compliance rates of 60–70%.^37^ Though this intervention is perhaps of greatest impact in preventing hospital-acquired infection in low-resource settings. Tesfaye *et al* observed hand hygiene compliance as low as 2.8% in Ethiopian delivery wards.^34^ Contributing factors—limited water, soap and time—mirrored our findings in Malawi, as did lower compliance at night.^34^

The implications of our findings for clinical practice and healthcare policy include the need to improve the availability of basic resources for infection prevention and for closer supervision and mentorship of HCWs by senior staff at facility level. Furthermore, our findings regarding the context for implementation had critical implications for the development and success of a complex intervention to improve infection prevention at the time of caesarean section in the study setting and later in a multicountry randomised controlled trial.^38^ We would recommend that future researchers and implementers conduct in-depth mixed-methods evaluations to understand the implementation context before seeking to effect change

## Conclusion

Our study demonstrates that adherence to recommended IP practice is determined by more than the availability of resources; the indirect impact of scarcity on staff motivation also played an important role. The study identified barriers and facilitators to general IP practices, the understanding of which was subsequently used in the selection and development of a complex behaviour change intervention to improve IP practices in the same facilities.

## Supplementary material

10.1136/bmjgh-2025-020210 online supplemental file 1

10.1136/bmjgh-2025-020210 online supplemental file 2

## Data Availability

Data are available upon reasonable request.
