# Supplementary material for: More than a lack of resources: a mixed-methods assessment of the barriers and facilitators to infection prevention practices in three low-resource maternity units in Malawi
Source: BMJ Glob Health. 2026 Sep 15;11(9):e020210. doi: 10.1136/bmjgh-2025-020210 (PMC13583800; doi:10.1136/bmjgh-2025-020210)
Supplement: Supplementary file 1 [file bmjgh-11-9-s001.docx]

**SUPPLEMENTARIES**

**Figure S1. Interaction of the components COM-B (Capability-Opportunity-Motivation) Model of Behaviour Change**

The COM-B and TDF are fully referenced in the accompanying manuscript.

Expansion of the COM-B model to include the theoretical domains framework is outlined below.

| **COM-B** |  | **Domain of TDF** | **Definition** |
| --- | --- | --- | --- |
| **Capability** | **Physical Capability** | **Physical Skills** | An ability or proficiency acquired through practice |
|  | **Psychological Capability** | **Knowledge** | An awareness of the existence of something |
|  |  | **Cognitive and interpersonal skills** | An ability to perform the necessary thought processes and social interactions required to carry out the behaviour |
|  |  | **Memory, Attention & Decision Processes** | The ability to retain information, focus selectively on aspects of the environment and choose between two or more alternatives |
|  |  | **Behavioural regulation** | Anything aimed at managing or changing objectively observed or measured actions |
| **Motivation** | **Reflective Motivation** | **Social/professional roles and identity** | A coherent set of behaviours and displayed personal qualities of an individual in a social or work setting |
|  |  | **Beliefs about capabilities** | Acceptance of the truth, reality, or validity about an ability, talent, or facility that a person can put to constructive use |
|  |  | **Optimism** | The confidence that things will happen for the best or that desired goals will be attained |
|  |  | **Beliefs about consequences** | Acceptance of the truth, reality, or validity about outcomes of a behaviour in a given situation |
|  |  | **Intentions** | A conscious decision to perform a behaviour or a resolve to act in a certain way |
|  |  | **Goals** | Mental representations of outcomes or end states that an individual wants to achieve |
|  | **Automatic Motivation** | **Reinforcement** | Increasing the probability of a response by arranging a dependent relationship, or contingency, between the response and a given stimulus |
|  |  | **Emotion** | A complex reaction pattern, involving experiential, behavioural, and physiological elements, by which the individual attempts to deal with a personally significant matter or event |
| **Opportunity** | **Physical opportunity** | **Environmental context and resources** | Any circumstance of a person’s situation or environment that discourages or encourages the development of skills and abilities, independence, social  competence, and adaptive behaviour |
|  | **Social opportunity** | **Social influences** | Those interpersonal processes that can cause individuals to change their thoughts, feelings, or behaviours |

Table S1. The Theoretical Domains Framework (TDF). COM-B = Capability-Opportunity-Motivation-Behaviour

**S2. Patient participant consent form**

**S3. Healthcare worker participant consent form**

**S4: Case Report Form: Quantitative data collection for women undergoing caesarean section**

**S5. Weekly Resource Availability Survey**

**S6. Vignette 1: For use in interviews with labour ward staff**

*Beatrice is a 21yr old who has her first baby at a local healthcare facility. She has a Caesarean section. After 10 days she returns to the facility with her wound gaping and signs of infection. She has peritonitis and is referred to the central hospital where she has a hysterectomy. She makes a good recovery…*

*Now I will tell you the story of Beatrice. I will tell you part of the story, then ask you to help me complete it by asking you questions about the characters in the story and what they would do…*

*Beatrice goes into labour and attends the health facility. When she gets there, she is met by a male nurse/midwife who will assess her. His name is Ernest. Ernest decides to perform a vaginal examination to determine if Beatrice is in labour or not…*

*Ernest does not wash his hands, but he puts on gloves. He gets sterile gauze from the container; he runs it under the tap and carries it to the bed where Beatrice is. Ernest finds that Beatrice is 5cm dilated. As Beatrice is in active labour, Ernest admits her to labour ward.*

*Welani, one of the other nurses is watching Ernest. She often sees him doing his examinations like this and worries about whether the patients will get infections…*

*Beatrice has a long labour and does not make good progress. Eventually, Gift (the clinician) decides that she should have a caesarean due to cephalopelvic disproportion. Gift remembers being involved in a study where the research team tried to encourage vaginal cleansing immediately before the caesarean to reduce the risk of endometritis. He thinks about suggesting this for Beatrice, but he’s not sure if the rest of the team will agree, so he doesn’t mention it.*

*Gift successfully completes the procedure. There is a sign on the wall of the theatre asking if the swabs and instruments have been counted, but nobody talks about this. There is also a poster from the World Health Organisation that talks about Surgical Checklists.*

*Beatrice is transferred to the postnatal ward soon after the surgery is finished. She makes a good recovery over the next few days and gets ready to go home.*

*As you know, Beatrice goes on to develop an infection and needs to have a hysterectomy. She makes a good recovery but can’t have any more children.*

**S7. Vignette 2: For use in interviews with theatre staff**

*Beatrice is a 21yr old who has her first baby at a local healthcare facility. She has a caesarean section. After 10 days she returns to the facility with her wound gaping and signs of infection. She has peritonitis and is referred to the central hospital where she has a hysterectomy. She makes a good recovery…*

*Now I will tell you the story of Beatrice. I will tell you part of the story, then ask you to help me complete it by asking you questions about the characters in the story and what they would do…*

*Beatrice is having her first baby. She has a long labour and does not make good progress. Eventually, Gift (the clinician) decides that she should have a caesarean section due to cephalo-pelvic disproportion. However, Beatrice is delayed going to theatre by several hours as there is another case which must go first. It is 6 hrs since the decision was made for caesarean and she now arrives in theatre. Gift is in theatre getting ready to perform the procedure.*

*Gift scrubs for the surgery using buckets and soap. Welani, the theatre nurse often sees that he is not careful with how he scrubs or places the drapes, and she worries about the patients getting infections.*

*Nelson is the anaesthetist. He knows this has been a long labour for Beatrice, and he thinks there is a high risk of infection. He remembers being involved in a study where the research team tried to encourage vaginal cleansing immediately before the caesarean section to reduce the risk of endometritis. He thinks about suggesting this for Beatrice, but he’s not sure if the rest of the team will agree, so he doesn’t mention it.*

*Gift proceeds with the Caesar. The baby cries soon after birth. The surgery is difficult and at some points, Gift is not sure if he can manage to save the uterus, but he does and closes the patient.*

*There is a sign on the wall of the theatre asking if the swabs and instruments have been counted, but nobody talks about this. There is also a poster from the World Health Organisation that talks about Surgical Checklists.*

*Beatrice goes to the postnatal ward. After that she goes home, before she returns with signs of infection which require her to have a hysterectomy.*

**S8. Heat maps of resource availability at study facilities**

S9. Main findings of participant observation summarised under domains of framework constructed from relevant infection prevention guidelines.

CS = caesarean section, WASH = water, sanitation and hygiene, VE = vaginal examination, HH = hand hygiene.

**S10. In-depth interview participants**

| **Site** | **Role** | **Number** | **Areas Worked** |
| --- | --- | --- | --- |
| **A** | Clinical Officer (Obstetrics) | 2 | Theatre, all maternity wards |
|  | Clinical Officer (Obstetrics) | 1 | Postnatal ward, (No surgery) |
|  | Intern Clinical Officer | 1 | Theatre, all maternity wards |
|  | Clinical Officer (Anaesthetics) | 1 | Theatre |
|  | Nurse/Midwife | 1 | Labour Ward |
|  | Nurse/Midwife | 1 | Postnatal Ward |
| **B** | Nurse/Midwife | 3 | Labour ward |
| **C** | Nurse/Midwife | 4 | Maternity wards |
|  | In charge Nurse/Midwife | 1 | Labour ward |
|  | Nurse/Midwife | 1 | Theatre, maternity wards |
|  | Clinical officer | 1 | Theatre, maternity wards |
