# Supplementary material for: More than a lack of resources: a mixed-methods assessment of the barriers and facilitators to infection prevention practices in three low-resource maternity units in Malawi
Source: BMJ Glob Health. 2026 Sep 15;11(9):e020210. doi: 10.1136/bmjgh-2025-020210 (PMC13583800; doi:10.1136/bmjgh-2025-020210)
Supplement: Supplementary file 2 [file bmjgh-11-9-s002.docx]

### BMJ Global Health Author Reflexivity Statement

Adapted from Morton, B., Vercueil, A., Masekela, R., Heinz, E., Reimer, L., Saleh, S., Kalinga, C., Seekles, M., Biccard, B., Chakaya, J., Abimbola, S., Obasi, A. and Oriyo, N. (2022), Consensus statement on measures to promote equitable authorship in the publication of research from international partnerships. Anaesthesia, 77: 264-276. <https://doi.org/10.1111/anae.15597>

| **Study conceptualisation** | |
| --- | --- |
| 1. How does this study address local research and policy priorities? | Reducing preventable maternal deaths is a top priority for the Malawi Ministry of Health (Gondwe et al, 2025)  Infection is the leading cause of maternal death in Malawi (Riches, 2025), as determined by data published on behalf of the Ministry of Health of Malawi and therefore infection prevention emerged as a policy priority during consultation with key stakeholders (Riches, 2025). |
| 2. How were local researchers involved in study design? | Local researchers were key protocol contributors in the supervision of this PhD research work by JR. LNM is a local Malawian health systems researcher with clinical background as a nurse and midwife and had a role in supervising the development of the qualitative aspects of the study desugb (participant observation and interviews). |
| **Research management** | |
| 3. How has funding been used to support the local research team(s)? | Funding for this study was provided by the NIHR and directly supported the work of the maternal health research group within MLW, Blantyre, Malawi as well as supporting the general overheads of the research institute. Funding for the study also supported a local research coordinator, local data collection officers and transcription team. |
| **Data acquisition and analysis** | |
| 4. How are research staff who conducted data collection acknowledged? | Although they do not fit international criteria for manuscript authorship, local data collection officers are directly named in the “Acknowledgements” section of the manuscript. |
| 5. How have members of the research partnership been provided with access to study data? | Data collected was summarised and presented to local research partners (senior staff at study sites) |
| 6. How were data used to develop analytical skills within the partnership? | Data collected was summarised and presented to local research partners (senior staff at study sites). An explanation of how data were analysed was provided.  Further to this, the local study coordinator was mentored by the researcher undertaking the study (JR) to further develop their analytical skills during the study. |
| **Data interpretation** | |
| 7. How have research partners collaborated in interpreting study data? | The study coordinator provided formal insight allowing the interpretation of all study data as findings were discussed with them during and following data collection, contributing to data analysis. This is explicitly acknowledged in the manuscript/. |
| **Drafting and revising for intellectual content** | |
| 8. How were research partners supported to develop writing skills? | Research partners were invited to comment and revise the manuscript prior to submission. |
| 9. How will research products be shared to address local needs? | The published manuscript will be disseminated locally at all levels of the health system to enable policy changes and review of practice. |
| **Authorship** | |
| 10. How is the leadership, contribution and ownership of this work by LMIC researchers recognised within the authorship? | They are named as authors (CK, LNM). Where they do not meet the criteria for authorship, they are acknowledged as contributors. |
| 11. How have early career researchers across the partnership been included within the authorship team? | Acknowledged as authors (CK and MO) |
| 12. How has gender balance been addressed within the authorship? | Gender balance within the authorship represents the roles undertaken by named authors. |
| **Training** | |
| 13. How has the project contributed to training of LMIC researchers? | The local study coordinator (CK) was mentored by the researcher undertaking the study (JR) to further develop their research skills and is now undertaking a masters degree. |
| **Infrastructure** | |
| 14. How has the project contributed to improvements in local infrastructure? | The project has highlighted the need for improvement in local infrastructure and findings will be disseminated. |
| **Governance** | |
| 15. What safeguarding procedures were used to protect local study participants and researchers? | Local research institution safeguarding policies were followed to ensure protection for local study participants and researchers. This was overseen by the research support unit at MLW as per protocol. |
